# Remarks from Dr. Bree on Disordered Respiration

**Published:** 1810-11

**Authors:** 


### *To the Editors of the Medical and Physical Journal.*

Gentlemen,

I was led by your criticism on Dr. Rees's observations on disorders of the stomach, to peruse his treatise. Dr. Rees has made use, at the latter end of his remarks, of a well-known work by Dr. Bree on disordered respiration, but I was rather surprised at his report of the opinions of that writer. Dr. Rees gives a quotation from this work, "*Not merely from his admiration of its merit, but because the author has no intention of attributing to the stomach that share in the production of the disease which he is persuaded it possesses.*" To this quotation now referred to, he adds many more from different parts of the work; as testimony *unintended* of the truth of the theory, that a dyspeptic state of the stomach causes asthma. It appears, however, on perusing the "Practical Inquiry," that Dr. Bree has no doubtful opinion on the subject, and the reader will there see fully proved what Dr. Rees shews in part, by quotations, "That Dyspepsia is a certain *predisposing* cause in all cases, and so frequently an *immediate* cause as to form one species of asthma."—Pract. Inq. p. 148.

A quotation that appears at pag. 189, of Dr. Rees's work, might have been given with more precision, as follows, viz. "Whenever dyspepsia prevails, there shall we find a fruitful opportunity of exciting the paroxysm of asthma. If it exist, unaccompanied by other causes, it may be said to occasion the *third species*; but this debility of the stomach must probably concur with other causes before the disease appears in the form of the first species."—Pract. Inq. 4th Edit. p. 145.

Treating of this complaint as a predisposing cause of asthma, asthma, Dr. Bree says—"*Sir J. Floyer considers this state of the stomach as secondary to the state of the lungs, and not as an immediate cause; but the contrary order is more probable, since asthma seldom appears without having been preceded by dyspepsia, though the latter frequently occurs unconnected with asthma. The power of digestion will certainly be additionally weakened whenever asthma is formed, and in this view dyspepsia may follow asthma; but it has previously been a cause of the disease, which reciprocally augments, and is itself aggravated by, this debility.*"

This must be the opinion of every observing Physician, if he carefully attend to causes of asthma, and reflect on his experience. But hitherto it remains a fact, not creditable to our science, that the authority of Floyer making dyspepsia in all cases a symptom of disordered lungs, is maintained in the practice, however ineffective, of most practitioners.

Under the head of third species of asthma, Dr. Bree says, "Acrimony in the stomach, and duodenum excites the associated effort of all the respiratory muscles which act by sympathy, as if the cause of offence peculiarly disturbed the lungs."—Pag. 208.

He gives instances in which disorder of the stomach was only indicated.—Pag. 209. And at the conclusion of the section his deduction is given from the whole. "It has appeared in the preceding sections, that convulsive asthma is caused by the irritation of some material in the lungs."

"We may conclude from the analogies, the reasoning, and the facts before us, that it may also be caused by irritation in some of the abdominal viscera, but particularly in the *stomach*."

It appears manifestly, therefore, that Dr. Bree "*intended*" to make the affection of the stomach a cause of asthma in all cases of that disease; that in a great majority of cases, which make the first species, dyspepsia is treated of, and considered as a predisposing cause; and that in numerous cases, classed under the third species, he considers it an immediate or proximate cause of asthma.

MEDICUS.

